# *De novo* protein structure determination by heavy-atom soaking in lipidic cubic phase and SIRAS phasing using serial synchrotron crystallography

**DOI:** 10.1107/S2052252518009223

**Published:** 2018-08-08

**Authors:** S. Botha, D. Baitan, K. E. J. Jungnickel, D. Oberthür, C. Schmidt, S. Stern, M. O. Wiedorn, M. Perbandt, H. N. Chapman, C. Betzel

**Affiliations:** a Institute of Biochemistry and Molecular Biology, Chemistry Department, University of Hamburg, Martin-Luther-King Platz 6, 20146 Hamburg, Germany; b Laboratory for Structural Biology of Infection and Inflammation, c/o DESY, Building 22a, Notkestrasse 85, 22607 Hamburg, Germany; c The Hamburg Centre for Ultrafast Imaging, Luruper Chaussee 149, 22761 Hamburg, Germany; d Xtal Concepts GmbH, Marlowring 19, 22525 Hamburg, Germany; e Center for Free-Electron Laser Science, Deutsches Elektronen-Synchrotron (DESY), Notkestrasse 85, 22607 Hamburg, Germany; f Department of Physics, University of Hamburg, Luruper Chaussee 149, 22761 Hamburg, Germany

**Keywords:** serial crystallography, SIRAS phasing, lipidic cubic phase, heavy-atom soaking, *de novo* protein structure determination

## Abstract

During the past few years, serial crystallography methods have undergone continuous development and serial data collection has become well established at high-intensity synchrotron-radiation beamlines and XFEL radiation sources. However, the application of experimental phasing to serial crystallography data has remained a challenging task owing to the inherent inaccuracy of the diffraction data. Here, a particularly gentle method for incorporating heavy atoms into micrometre-sized crystals utilizing lipidic cubic phase (LCP) as a carrier medium is reported. Soaking in LCP prior to data collection offers a new, efficient and gentle approach for preparing heavy-atom-derivative crystals directly before diffraction data collection using serial crystallography methods. This approach supports effective phasing by utilizing a reasonably low number of diffraction patterns. Using synchrotron radiation and exploiting the anomalous scattering signal of mercury for single isomorphous replacement with anomalous scattering (SIRAS) phasing resulted in high-quality electron-density maps that were sufficient for building a complete structural model of proteinase K at 1.9 Å resolution using automatic model-building tools.

## Abstract 2

Lipid cubic phase supplemented with mercury was used to create a heavy-atom derivative of microcrystals for direct SIRAS phasing using serial millisecond crystallography.

## 1. Introduction   

Serial femtosecond crystallography (SFX) at XFEL radiation sources and serial crystallography at synchrotron-radiation sources (serial millisecond crystallography; SMX) are relatively new approaches for the collection of diffraction data for the structure determination of biological macromolecules. Methods, software and procedures have been undergoing continuous development over the last decade (Chapman *et al.*, 2011 ▸; Boutet *et al.*, 2012 ▸; Schlichting, 2015 ▸; Cheng *et al.*, 2017 ▸). Suspensions of micrometre- or nanometre-sized crystals are streamed across the X-ray beam at a free-electron laser (XFEL) or high-intensity synchrotron-radiation source and diffraction patterns are collected in a diffraction-before-destruction approach (Neutze *et al.*, 2000 ▸). The diffraction patterns of thousands of individual crystals in random orientations are collected and the intensities are integrated using a Monte Carlo approach (Kirian *et al.*, 2010 ▸; White *et al.*, 2012 ▸). Serial diffraction data have been shown to be of sufficient quality for *de novo* phasing approaches (Barends *et al.*, 2014 ▸; Nass *et al.*, 2016 ▸; Yamashita *et al.*, 2015 ▸, 2017 ▸; Nakane *et al.*, 2016 ▸; Gorel *et al.*, 2017 ▸). However, systematic inaccuracies and variances in the data resulting from experimental factors, such as the wide spectral distribution of the XFEL as well as changes in the sample-to-detector distance when exchanging the sample-delivery nozzle and variations in the size of the microcrystal distribution or liquid-jet width, have been shown to severely hamper phasing attempts using heavy-atom data sets (Nass *et al.*, 2016 ▸). This has so far been overcome by using a large number of diffraction patterns, and Yamashita *et al.* (2015 ▸) demonstrated that single-wavelength anomalous diffraction (SAD) phasing with approximately 80 000 patterns as well as single isomorphous replacement with anomalous scattering (SIRAS) phasing with approximately 20 000 patterns were feasible. Nass *et al.* (2016 ▸) showed that by systematically addressing some of the aforementioned in­accuracies, SAD phasing using gadolinium was possible with only approximately 10 000 patterns in the case of lysozyme, and native sulfur SAD was successfully applied to phase the crystal structure of thaumatin with approximately 125 000 images. In a different study, Nakane *et al.* (2016 ▸) systematically reduced the number of images used for successful SIRAS phasing of the membrane protein bacteriorhodopsin using an iodine-labelled detergent heavy-atom additive and determined the minimal number of patterns required to obtain meaningful phases. They showed that 12 000 patterns were sufficient for successful SIRAS phasing, and a further reduction in the number of images required was achieved by a combination of single isomorphous replacement (SIR) and SIRAS (Nakane *et al.*, 2016 ▸).

One of the hurdles that has been addressed extensively over the past decade is how to produce a steady stream of microcrystals passing through the X-ray beam. The initially developed method for SFX experiments, which is still extensively used, is the delivery of crystals *via* a liquid jet (Chapman *et al.*, 2011 ▸; Boutet *et al.*, 2012 ▸). However, until now liquid-jet delivery has required a relatively large amount of crystal suspension, with a large number of crystals going to waste without contributing a diffraction pattern. Furthermore, this delivery method has severe shortcomings for synchrotron-radiation sources owing to the jet speed of many metres per second not placing the crystals in the beam long enough to yield useful diffraction (Beyerlein *et al.*, 2015 ▸). Therefore, other sample-delivery techniques for SFX were developed with the aim of reducing the amount of crystal suspension required to collect a complete data set. Subsequently, methods compatible with atmospheric operation were also adapted for synchrotron-radiation beamlines and it was shown that serial diffraction data collected from crystals in the micrometre size range have sufficient quality to be used for phasing calculations. Serial crystallography has been adapted to synchrotron application during recent years (Beyerlein *et al.*, 2017 ▸; Botha *et al.*, 2015 ▸; Gati *et al.*, 2014 ▸; Stellato *et al.*, 2014 ▸; Weinert *et al.*, 2017 ▸) and the feasibility of phasing serial synchrotron data collected at a synchrotron using MIRAS has been demonstrated (Botha *et al.*, 2015 ▸) as well as using SAD (Weinert *et al.*, 2017 ▸).

Here, we show that diffraction data collection using a single derivative was sufficient for *de novo* structure determination of the model system proteinase K. Experimental phases were determined by SIRAS phasing of the serially collected data. The resulting electron-density maps were of good quality, allowing automatic model building of the entire structure apart from the two terminal alanine residues. Furthermore, the crystals used in this study were all grown natively and the derivative diffraction data were collected by introducing these crystals into lipidic cubic phase (LCP) containing mercury. A lipidic cubic phase injector was used to stream micrometre-sized proteinase K crystals embedded in lipidic cubic phase across the X-ray beam in a serial crystallography approach. Data were collected by continuous, shutterless operation at room temperature. This procedure was sufficient to reach an occupancy of 0.4 for both possible (mutually exclusive) mercury sites after minutes of *in situ* soaking and proved to be a very gentle way of obtaining derivative crystals, with almost no variation in the unit-cell parameters of the derivative crystals compared with the native unit-cell parameters. The data were subsequently phased using SIRAS and the model could be built using automatic model-building tools.

## 2. Materials and methods   

**Figure 1:**
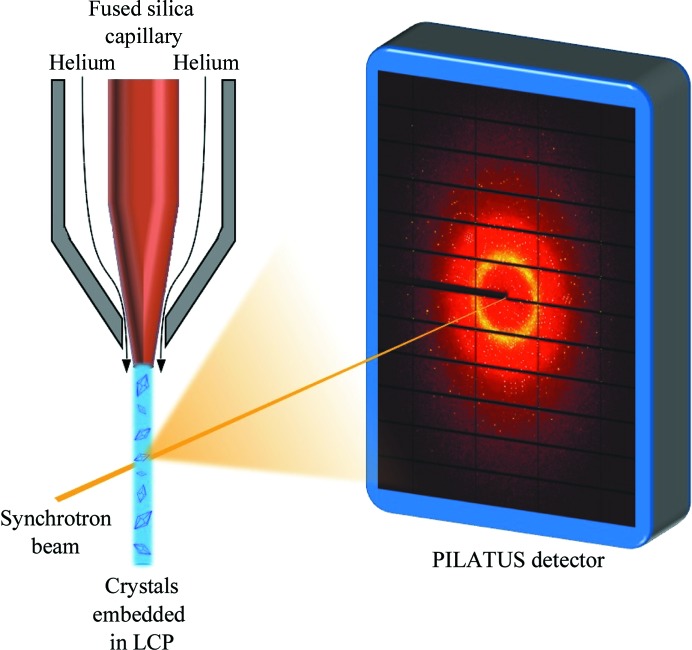
Experimental crystal-delivery setup.

Proteinase K crystals were grown using the batch method with initial conditions taken from Betzel *et al.* (2001 ▸). Proteinase K was obtained from Merck KGaA and the protein was dissolved to 20 mg ml^−1^ in protein buffer consisting of 50 m*M* Tris–HCl pH 7.0, 10 m*M* CaCl_2_, resulting in a final protein concentration of 20 mg ml^−1^. 50 µl protein solution was mixed with 15 µl precipitant solution consisting of 2 *M* NaNO_3_ and a further 80 µl protein buffer. Crystals of dimensions 5 × 5 × 10 µm grew overnight and were washed with crystal-storage solution consisting of 75%(*v*/*v*) buffer and 25%(*v*/*v*) precipitant. LCP was obtained using dl-α-monoolein, 9.9 MAG, obtained from Fluka and crystal-storage solution. Monoolein was mixed with crystal-storage solution in a 60:40%(*w*:*v*) ratio using coupled Hamilton syringes (Cheng *et al.*, 1998 ▸) and the respective volumes were then adjusted until a clear and isotropic phase formed. For the mercury-containing LCP, the crystal-storage solution was supplemented with 1 mg ml^−1^ HgCl_2_ prior to mixing with the lipid. The mercury concentration was selected to guarantee a high abundance of mercury in the LCP compared with the protein concentration. For both the derivative and the native LCP material, 8 µl of native proteinase K crystal pellet (leaving the crystal suspension to settle into a pellet overnight) was embedded into 20 µl of the respective LCP immediately prior to injection, as described in Botha *et al.* (2015 ▸), and the crystal-containing LCP was transferred to the sample reservoir of the injector (Weierstall *et al.*, 2014 ▸; Liu *et al.*, 2014 ▸; Botha *et al.*, 2015 ▸). A nozzle with a 50 µm internal diameter capillary was used to inject the crystals, and the helium pressure was set to 1655–3240 kPa. The flow rate on the HPLC pump varied from 2.5 to 4 µl min^−1^ with an associated pressure of 255–331 kPa, corresponding to a sample flow rate of 73–113 nl min^−1^. After passing the X-ray beam, the sample was collected in a catcher as described previously (Weierstall *et al.*, 2014 ▸; Liu *et al.*, 2014 ▸) and disposed of as appropriate. A schematic overview of the data-collection setup is shown in Fig. 1 ▸. Diffraction data were collected on the P11 beamline at PETRA III (Burkhardt *et al.*, 2016 ▸), Deutsches Elektronen Synchrotron (DESY) in May 2016. The PILATUS 6M detector was operated in shutterless mode at 20 Hz. The exposure time per image was set at 20 ms, the detector distance was kept constant at 200 mm and the beam energy was set to 12 keV. The hit rate was monitored online using *OnDA* (Mariani *et al.*, 2016 ▸). Hit finding, indexing and integration were performed using *CrystFEL* v.0.6.1 (White *et al.*, 2012 ▸) and phasing using the *SHELX* package v.2016/1 (Usón & Sheldrick, 1999 ▸; Sheldrick, 2002 ▸, 2010 ▸). Subsequent density modification was performed with *DM* (Cowtan, 1994 ▸) and the electron density calculated from the phased data was passed to the automatic model-building program *ARP*/*wARP* (Langer *et al.*, 2008 ▸). The resulting structure was refined using alternate cycles of *REFMAC*5 (Murshudov *et al.*, 2011 ▸) in the *CCP*4 suite (Winn *et al.*, 2011 ▸) and manual refinement using *Coot* (Emsley *et al.*, 2010 ▸). To test the robustness of the data, the raw integrated intensities of the native and derivative data were also passed to *Auto-Rickshaw* (Panjikar *et al.*, 2005 ▸, 2009 ▸) for automatic phasing and model building.

## 3. Results   

For the native data set, a total of 162 702 images were collected in 6 h. The data were submitted to *CrystFEL* without prior hit finding and 28 674 patterns could be indexed by *CrystFEL* using a threshold setting of 50 and a minimum signal-to-noise ratio of 2.5 for peak detection. The crystals diffracted to a resolution of 1.89 Å. The data-collection and refinement statistics are summarized in Table 1 ▸. For the mercury derivative 397 626 images were recorded in 15.5 h, of which 64 665 could be indexed using the same peak-finding parameters as for the native data, indicating that the mercury-supplemented sample had no significant impact on the background scattering. Additionally, no decrease in crystal quality or resolution was observed for the derivative data. Both data sets were processed in space group *P*4_3_2_1_2, with unit-cell parameters *a* = *b* = 67.6, *c* = 107.4 Å, α = β = γ = 90°. These unit-cell parameters were determined by fitting a Gaussian distribution to the individually determined unit-cell parameters for each indexed diffraction pattern for both serial data sets with the *cell-explorer* program from *CrystFEL*. The *partialator* routine from *CrystFEL* was then used to scale and post-refine the reflections, also accounting for the partiality of the respective reflections, in three iterations using the unity model.

**Figure 2:**
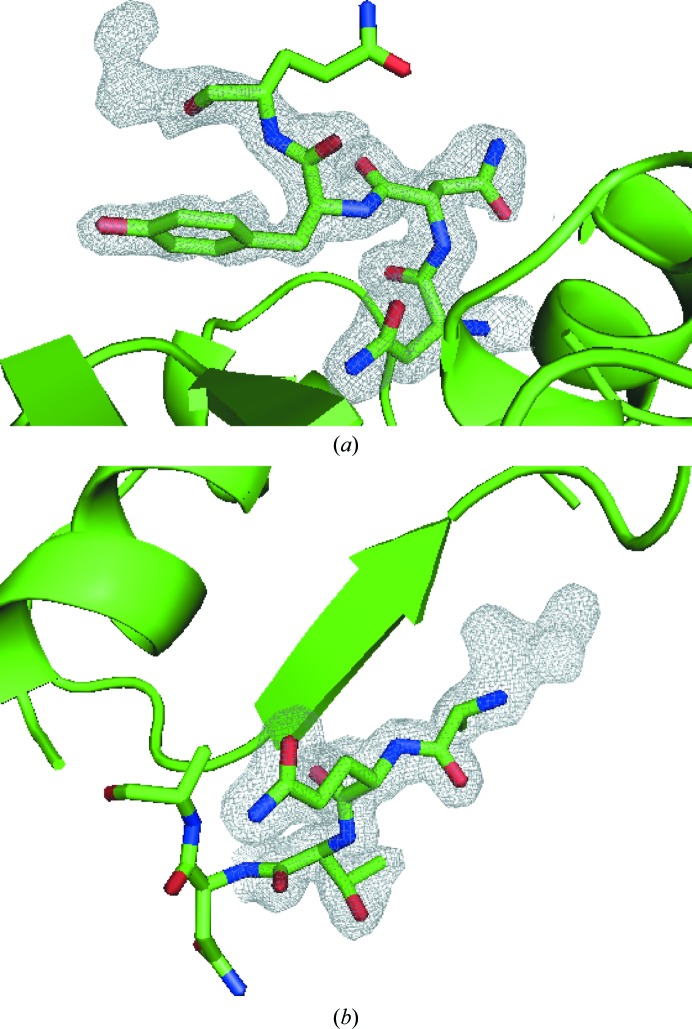
2*F*_o_ − *F*_c_ electron-density maps of (*a*) the N-terminal and (*b*) C-terminal alanines contoured at 1.0σ after ten cycles of model building with *ARP*/*wARP*.

**Figure 3:**
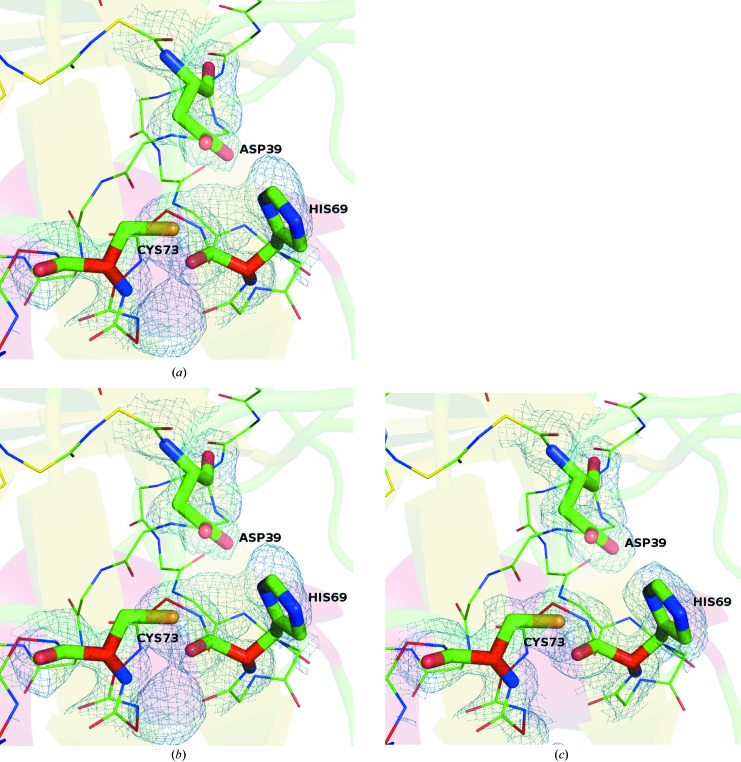
2*F*_o_ − *F*_c_ electron-density maps calculated using the phases after applying (*a*) *SHELXE*, (*b*) *DM* and (*c*) the final refined phases, all contoured at 1.0σ and overlaid with the final refined structure. The active site is shown and the residues forming the catalytic triad are labelled.

**Figure 4:**
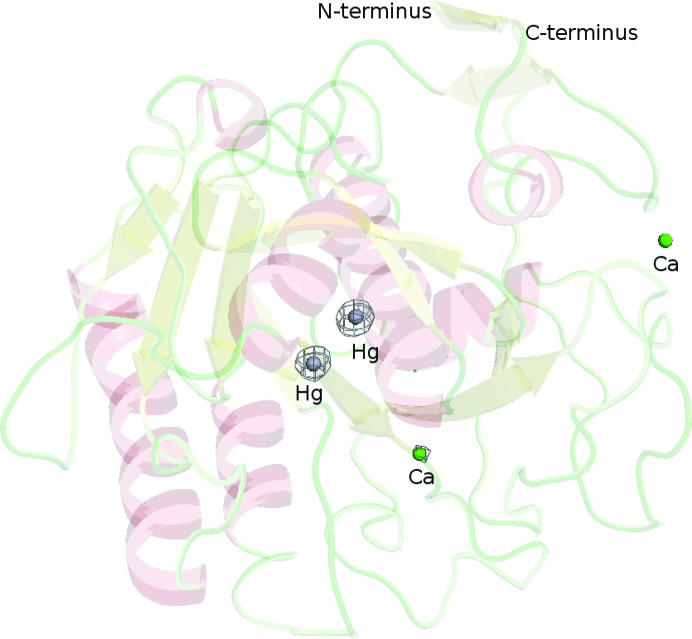
Cartoon plot of proteinase K with the anomalous difference density map calculated from all 64 665 indexed derivative images and the phases from the final refined model contoured at 5.0σ. The two Hg atoms (silver spheres) covalently bind to Cys73. The two green spheres correspond to the two bound calcium ions.

The processed diffraction data were prepared for SIRAS phasing using *SHELXC* and *SHELXD*, and subsequently two heavy-atom sites were found. These correspond to the mercury-binding sites of proteinase K reported previously by Gourinath *et al.* (2001 ▸). Subsequently, 20 cycles of phasing and density modification were performed by *SHELXE*; the best solution had a CC_all_ of 44.9 and a CC_weak_ of 34.40, and a PATFOM of 64.44 was obtained. The resulting phases were subjected to a further ten cycles of density modification by *DM*, and after inspecting the original and inverse density maps the original hand was deemed to be correct, with a resulting improvement in the FOM from 0.66 to 0.84. This density was then submitted to automatic model building using *ARP*/*wARP*, providing the protein sequence. Ten rounds of *ARP*/*wARP* resulted in a structure that was 99.28% complete, with only the two terminal alanine residues not being automatically built. The results were inspected in *Coot* and clear electron density at both termini indicated the missing alanines, as shown in Fig. 2 ▸. Alternate cycles of manual refinement in *Coot* and automatic refinement with *REFMAC*5 resulted in a well refined structure including 120 solvent molecules and two Ca^2+^ ions, with a final *R*_work_ and *R*_free_ of 14.23% and 17.14%, respectively. The 2*F*_o_ − *F*_c_ maps calculated after phasing with *SHELXE*, after solvent flattening with *DM* and the final refined map are shown in Fig. 3 ▸, all contoured at 1.0σ and overlaid with the final refined structure. The anomolous density map calculated from all 64 665 indexed derivative images and the phases from the final refined model contoured at 5σ is shown in Fig. 4 ▸.

**Figure 5:**
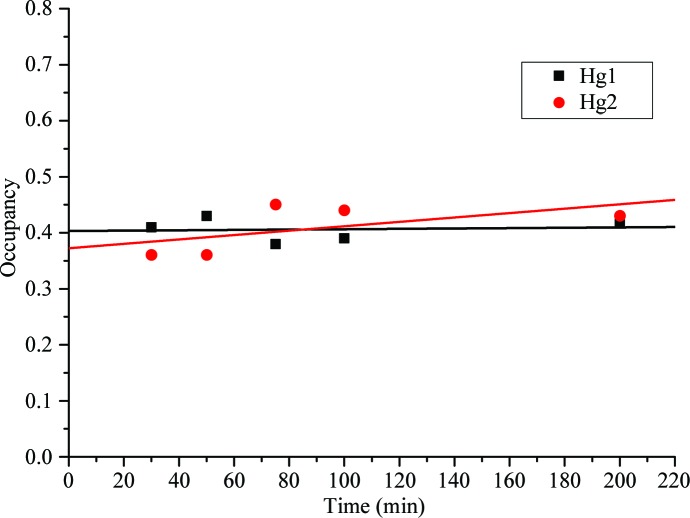
Temporal evolution of the refined occupancies of the Hg atoms. Data collected in the time intervals 7–30, 30–50, 50–75, 75–100 and 100–150 min and images taken after 150 min were processed individually and the occupancies of the Hg atoms were refined.

To further determine the robustness of these data for phasing, the raw native and derivative data from *CrystFEL* were submitted to the EMBL-HH automated crystal structure determination platform *Auto-Rickshaw*. This resulted in a structure that was 98.9% complete with only one missing residue compared with phasing the data manually, demonstrating the high phasing quality of these data. The structure in this case was refined to a final *R*_work_ and *R*_free_ of 15.69% and 19.52%, respectively. Furthermore, the derivative data were split into different soaking-time bins (7–30, 30–50, 50–75, 75–100 and 100–150 min) and the occupancy of the Hg atoms was refined. From this analysis no significant increase in the overall Hg-atom occupancies was observed during the duration of the measurements (Fig. 5 ▸), corresponding to a very efficient diffusion of mercury through the LCP and into the crystals. It can therefore be concluded that this method is a very simple and effective method of soaking heavy atoms into crystals without having to handle the usually more fragile derivative crystals after soaking, rendering it a very gentle method of introducing heavy atoms into crystals.

When using only 12 000 randomly selected patterns (5000 native and 7000 derivative with multiplicities of 41.8 and 64.9, respectively, or 4000 native and 8000 derivative with multiplicities of 33.3 and 73.4, respectively), the phases obtained from *SHELXE* were sufficient for *ARP*/*wARP* to successfully build over 90% of the structure in ten cycles. A summary of the data statistics is given in Tables 2 ▸ and 3 ▸. A further reduction in the number of images to 11 000 images in total (native/derivative: 3000/8000, 4000/7000, 5000/6000 or 6000/5000) was sufficient for substructure determination, but the resulting quality of the electron-density maps was no longer sufficient for automatic building of the structure with *ARP*/*wARP*. A total of 12 000 images, when assuming a realistic combined hit and indexing rate of 30%, could be collected in as little as 30 min on a synchrotron-radiation source beamline such as P11 at PETRA III, being limited only by the 25 Hz readout rate of the PILATUS detector. Furthermore, crystal hit rates of 100% have become increasingly common as indexing algorithms undergo improvement, meaning that increasing the crystal concentration would result in even faster data collection.

Yamashita *et al.* (2015 ▸) investigated SIRAS phasing of a mercury derivative of luciferin-regenerating enzyme using serial data collected at the SACLA XFEL source in Japan, and reported that SIRAS phasing could be successfully applied when using 10 792 native images with a multiplicity of 222.2 and 10 000 derivative images with a multiplicity of 106.2. When considering both the number of images as well as the multiplicity required for successful SIRAS phasing, our data collected at a synchrotron-radiation source show a clear reduction in both the number of patterns required as well as the associated multiplicity of the data. A further study of applying SIRAS phasing, also to data collected at the SACLA XFEL radiation source, reported that approximately 12 000 diffraction patterns were sufficient for successfully phasing bacteriorhodopsin using an iodine-labelled detergent heavy-atom derivative (Nakane *et al.*, 2016 ▸). The paper, however, only provides data statistics for the total data collected during the study and not for the reduced image-number subsets, and therefore does not allow a direct comparison. A recent study by Weinert *et al.* (2017 ▸) demonstrated successful SAD phasing of a G-protein-coupled receptor using a setup very similar to that used in this study. Using 186 688 indexed images with a multiplicity of 1945.2 and 100 cycles of *SHELXE* phasing, density modification and chain tracing yielded sufficient phases with a resulting map that allowed manual improvement of the model. In serial crystallography the integrated diffraction intensities are determined by averaging over a large number of still diffraction images using Monte Carlo integration. Despite the resulting data being accurate enough for structure solution using molecular replacement, direct phasing methods have only very rarely been successfully employed for serially collected data at an XFEL radiation source (Barends *et al.*, 2014 ▸; Yamashita *et al.*, 2015 ▸, 2017 ▸; Nakane *et al.*, 2016 ▸; Nass *et al.*, 2016 ▸; Gorel *et al.*, 2017 ▸; Weinert *et al.*, 2017 ▸). Furthermore, the applicability of *de novo* phasing methods to serially collected data from synchrotron-radiation sources has been even less widely explored, with the only attempts being undertaken in 2015 (Botha *et al.*, 2015 ▸), where lysozyme was phased by MIRAS using an iodide derivative as well as a gold derivative, and in 2017 (Weinert *et al.*, 2017 ▸), where phases could be obtained applying native SAD. To our knowledge, we report here the first time that SIRAS has successfully been applied to serially collected data at a synchrotron-radiation source, which could therefore be directly compared with the application of SIRAS phasing to SFX data.

## 4. Conclusions   

We have shown that native protein crystals can be derivatized directly in LCP prior to data collection, and that this is an effective yet gentle approach for introducing heavy atoms into protein crystals and mitigates the need for handling fragile crystals after soaking. Serial diffraction data were collected to high resolution from these derivatized, micrometre-sized crystals, and *de novo* phases could be calculated using SIRAS phasing from as few as 12 000 diffraction patterns. Furthermore, these phases were sufficient to autobuild almost the entire structure to a resolution of 1.90 Å using automatic model-building tools. Finally, the data were collected using conventional synchrotron radiation despite the use of microcrystals, and did not require scarcely available (until very recently) XFEL beam time. We conclude that this approach for introducing heavy atoms into native, micrometre-sized protein crystals combined with serial data collection at a synchrotron-radiation source is a powerful new method for solving protein structures by *de novo* phasing. Therefore, this study successfully bridges the gap between MIRAS and SAD, the only successful phasing attempts that have been demonstrated applying SMX to date.

## Figures and Tables

**Table 1:** Data-collection and refinement statistics for the native and mercury-derivative proteinase K data sets Values in parentheses are for the outer shell.

	Native data	Derivative data
Wavelength (Å)	1.033	1.033
No. of indexed images	28674	64665
Resolution range (Å)	19.91–1.90 (1.93–1.90)	19.91–1.89 (1.92–1.89)
Space group	*P*4_3_2_1_2	*P*4_3_2_1_2
*a*, *b*, *c* (Å)	67.6, 67.6, 107.4	67.6, 67.6, 107.4
α, β, γ (°)	90.0, 90.0, 90.0	90.0, 90.0, 90.0
Total reflections	8029942 (34674)	21365495 (45300)
Unique reflections	37456 (3731)	38258 (3824)
Multiplicity	214.6 (9.4)	558.6 (11.9)
Completeness (%)	99.90 (99.04)	99.97 (99.71)
Mean *I*/σ(*I*)	10.06 (2.83)	15.89 (3.10)
*R*_split_ (%)	8.66 (38.43)	5.68 (35.99)
CC*	0.998 (0.921)	0.999 (0.921)
CC_ano_	0.04	0.14
*R*_work_/*R*_free_ (%)	14.23/17.14	
No. of atoms		
Total	2199	
Macromolecules	2077	
Heteroatoms	2	
Waters	120	
R.m.s.d., bonds	0.019	
R.m.s.d., angles	1.784	
Coordinate error (Å)		
Luzzati	0.126	
Maximum likelihood	0.077	
Ramachandran favoured (%)	99.64	
Ramachandran outliers (%)	0.36	
Average *B* factor (Å^2^)		
Overall	16.6	
Protein	16.4	
Solvent	26.0	

**Table 2:** Data-collection statistics for subsets of native proteinase K data Values in parentheses are for the outer shell.

	6000 images	5000 images	4000 images	3000 images
Wavelength (Å)	1.033	1.033	1.033	1.033
Resolution range (Å)	19.91–2.04 (2.11–2.04)	19.91–2.04 (2.11–2.04)	19.91–2.04 (2.11–2.04)	19.91–2.04 (2.11–2.04)
Space group	*P*4_3_2_1_2	*P*4_3_2_1_2	*P*4_3_2_1_2	*P*4_3_2_1_2
*a*, *b*, *c* (Å)	67.6, 67.6, 107.4	67.6, 67.6, 107.4	67.6, 67.6, 107.4	67.6, 67.6, 107.46
α, β, γ (°)	90.0, 90.0, 90.0	90.0, 90.0, 90.0	90.0, 90.0, 90.0	90.0, 90.0, 90.0
Total reflections	1605620 (25039)	1339372 (21218)	1065881 (18213)	801425 (14459)
Unique reflections	32117 (3211)	32117 (3211)	32117 (3211)	32117 (3211)
Multiplicity	50.0 (7.9)	41.8 (6.7)	33.3 (5.9)	25.2 (4.9)
Completeness (%)	99.93 (99.28)	99.81 (98.16)	99.67 (96.95)	99.20 (92.84)
Mean *I*/σ(*I*)	5.35 (2.81)	4.96 (3.23)	4.49 (3.09)	4.03 (3.57)
*R*_split_ (%)	17.14 (41.84)	18.73 (44.41)	20.93 (46.02)	24.30 (49.58)
CC*	0.987 (0.903)	0.985 (0.892)	0.982 (0.879)	0.974 (0.868)
CC_ano_	0.03	0.03	0.02	0.02

**Table 3:** Data-collection statistics for subsets of derivative proteinase K data Values in parentheses are for the outer shell.

	8000 images	7000 images	6000 images	5000 images
Wavelength (Å)	1.033	1.033	1.033	1.033
Resolution range (Å)	19.91–2.04 (2.11–2.04)	19.91–2.04 (2.11–2.04)	19.91–2.04 (2.11–2.04)	19.91–2.04 (2.11–2.04)
Space group	*P*4_3_2_1_2	*P*4_3_2_1_2	*P*4_3_2_1_2	*P*4_3_2_1_2
*a*, *b*, *c* (Å)	67.6, 67.6, 107.4	67.6, 67.6, 107.4	67.6, 67.6, 107.4	67.6, 67.6, 107.4
α, β, γ (°)	90.0, 90.0, 90.0	90.0, 90.0, 90.0	90.0, 90.0, 90.0	90.0, 90.0, 90.0
Total reflections	2348178 (18845)	2071662 (16608)	1753861 (14174)	1474620 (11560)
Unique reflections	32117 (3211)	32117 (3211)	32117 (3211)	32117 (3211)
Multiplicity	73.4 (6.1)	64.9 (5.5)	55.1 (4.8)	46.6 (4.2)
Completeness (%)	99.60 (95.98)	99.43 (94.27)	99.11 (91.22)	98.54 (85.92)
Mean *I*/σ(*I*)	6.82 (5.51)	6.26 (3.27)	6.06 (5.58)	5.53 (4.05)
*R*_split_ (%)	13.86 (42.18)	14.65 (43.10)	15.73 (45.16)	16.92 (47.85)
CC*	0.992 (0.907)	0.991 (0.905)	0.989 (0.892)	0.988 (0.885)
CC_ano_	0.07	0.08	0.07	0.02
